# A Qualitative Study to Understand the Potential Efficacy of an Information-Based Sugar Reduction Intervention among Low Socioeconomic Individuals in the UK

**DOI:** 10.3390/ijerph16030413

**Published:** 2019-01-31

**Authors:** Hannah Forde, Emma Solomon-Moore

**Affiliations:** 1 UKCRC Centre for Diet and Activity Research (CEDAR), MRC Epidemiology Unit, University of Cambridge School of Clinical Medicine, Box 285 Institute of Metabolic Science, Cambridge Biomedical Campus, Cambridge CB2 0QQ, UK; 2 Centre for Exercise, Nutrition and Health Sciences, School for Policy Studies, University of Bristol, 8 Priory Road, Bristol BS8 1TZ, UK; 3 Department for Health, University of Bath, Claverton Down, Bath BA2 7AY, UK; e.solomon-moore@bath.ac.uk

**Keywords:** sugar, consumption, behaviour, reduction, socioeconomic status, health, intervention, qualitative

## Abstract

Sugar consumption in the UK consistently exceeds recommendations, despite the association it has with poor health outcomes. Low socioeconomic groups are most likely to over-consume sugar, which could exacerbate existing health disparities. Various interventions attempt to reduce the amount of sugar consumed, but their effectiveness is still unclear. This study qualitatively explored the sugar consumption behaviours of individuals experiencing food poverty, and examined how an information-based sugar reduction intervention might influence these behaviours. Eight clients and six volunteers from a food bank in Bristol (UK) completed semi-structured, one-to-one interviews that were thematically analysed. Food bank clients appeared to heavily consume sugar, with little understanding of the associated health effects and limited awareness of the intervention. Consumption behaviours were particularly influenced by personal and psychological factors, such as mental health; in addition to social factors, like familial behaviours and food access issues. It emerged that food bank clients’ often-challenging personal circumstances were likely to promote their sugar consumption. Making intervention materials visually appealing and easily comprehendible were found to be important for improving an intervention’s reception. Recommendations were developed to improve the efficacy of similar information-based sugar reduction interventions among socioeconomically deprived groups.

## 1. Introduction

Every year non-communicable diseases are responsible for 40 million deaths worldwide [1], most of which are attributable to four risk factors [2]. Of those factors, the contribution of poor diet is greater than the combined influence of alcohol, tobacco and physical inactivity [2,3]. Poor diet is partly attributable to “profound changes in our relationship with food” experienced over the last 40 years ([4], p. 5). This includes consumption of sugar which, across all ages, exceeds the Scientific Advisory Committee on Nutrition’s recommendation that free sugars should provide no more than 5% of daily total energy intake for those aged 2 years and over [5,6]. For example, the National Diet and Nutrition Survey combined across 2014/15 and 2015/16 show that mean intake of free sugars as a percentage of total energy intake for adults aged 19 to 64 are more than double this recommendation (11.2%) [6]. True consumption may even exceed these values, as some research suggests that consumption of high fat and high sugar foods are often under-reported [7]. The main sources of free sugars in these adults ranged from ‘sugar, preserves and confectionery’ (25%), to ‘cereal and cereal products’ (24%), and ‘non-alcoholic beverages’ (21%) [6]. This trend for over-consuming sugar is problematic because it increases the risk of overweight [8], type 2 diabetes [9], cardio-metabolic risks [10,11], poor oral health [12,13,14], and some cancers [15,16]. 

Variations in sugar intake by socioeconomic status (SES) elevate the importance of addressing the trend for heavy sugar consumption. Measures of sugar consumption operationalise several definitions of sugar, including free sugars, “all monosaccharides and disaccharides added to foods by the manufacturer, cook or consumer, plus sugars naturally present in honey, syrups and unsweetened fruit juices” ([5], p. 9); and non-milk extrinsic sugars (NMES), which are, “sugars not contained within the cellular structure of a food except lactose in milk and milk products “([5], p. 18). In earlier National Diet and Nutrition Surveys combined across 2008/09 to 2011/12, the mean intake of NMES – the former sugar definition operationalised by the UK government—were greater in the lowest income quintile compared with the highest [17]. Similarly, low-income respondents consumed more NMES than the general population in the Low Income Diet and Nutrition Survey (2003–2005) [18]. Thus, to avoid widening existing and persistent health disparities [19], it is imperative to lessen sugar consumption among low socioeconomic status (LSES) groups. 

Numerous interventions are attempting to kerb the population’s sugar consumption. These range from “upstream” structural interventions [3], like the UK Soft Drinks Industry Levy [20], to “downstream” individual-level interventions, such as the Change 4 Life campaign [21]. According to existing literature, whether these interventions are effective remains unclear [22], so it is unsurprising that there is much debate about which approach to sugar reduction interventions is best. The epidemiologist, Geoffrey Rose, famously advocated using population-level approaches in favour of targeting high-risk individuals [3,23], as there is some evidence of upstream interventions achieving larger, more equitable improvements to population health, known as an “effectiveness hierarchy” [24]. The degree of recipient “agency”, or personal resource, demanded by an intervention might be one mechanism underpinning this effectiveness hierarchy [25]. Agency suggests that upstream interventions requiring less individual input are more likely to be effective overall, and among LSES populations who are often less able to contribute substantial personal resource [25]. On this basis, public health specialists are increasingly in favour of whole-system, upstream interventions, notably advocated by the Secretary of State for Health and Social Care in their policy paper on preventative healthcare [26].

So why are high-agency, information-based interventions still pursued? Two key reasons are that they are most acceptable to those enacting and receiving interventions [25], and they are less likely to be circumvented by food and drink manufacturers and retailers than lower-agency policies [27]. Given that existing literature identifies the need to evaluate interventions in a broader range of contexts and with differentiation in sub-populations [22], the focus should then turn to ensuring interventions are relevant, understood, and well received by LSES populations that stand to benefit most from consuming less sugar. Numerous factors contribute towards fully understanding the receptiveness of LSES groups to sugar reduction interventions. Behavioural change theories, such as the Health Belief Model [28], suggest that the effectiveness of information-based interventions might depend on understanding determinants of consumption [29]. Individual health attitudes, which underlie socioeconomic gradients in behaviour [30], might also affect how such health information is received. For example, LSES is associated with lower health consciousness, stronger fatalistic health beliefs, and less thinking about the future [31]. An individual’s ability to obtain, process and understand basic health information and access services required to make appropriate health decisions, known as “health literacy” [32], is also likely to contribute towards intervention efficacy. Improving understanding of how these factors manifest in LSES individuals has the potential to help future information-based interventions prove effective for them.

At a time where overweight and obesity are critical public health issues, it is easy to forget the almost contradictory challenge of increasing food insecurity for certain populations in the UK [33]. Increasing food insecurity has been met with a rise in emergency food support [33], often provided by food banks who issue food parcels at minimal or no cost [34]. A wealth of reasons forces an individual into food insecurity. In addition to low income and changes to social security [35], debt, addiction, family breakdown, and low purchasing power also contribute to food bank usage [36,37]. Food bank clients also often face poor physical and mental health [36]. While there is mixed evidence for the dietary quality of food bank offerings [34,38,39], their clients are uniquely placed to provide insight into LSES sugar consumption behaviours.

This study aimed to qualitatively explore sugar consumption among LSES individuals residing in the UK, to understand more about the factors that influence sugar consumption behaviour, as well as their knowledge of the associated health effects of sugar consumption, and reception of information-based sugar reduction interventions. 

## 2. Materials and Methods

### 2.1. Design and Sampling

**Figure 1:**
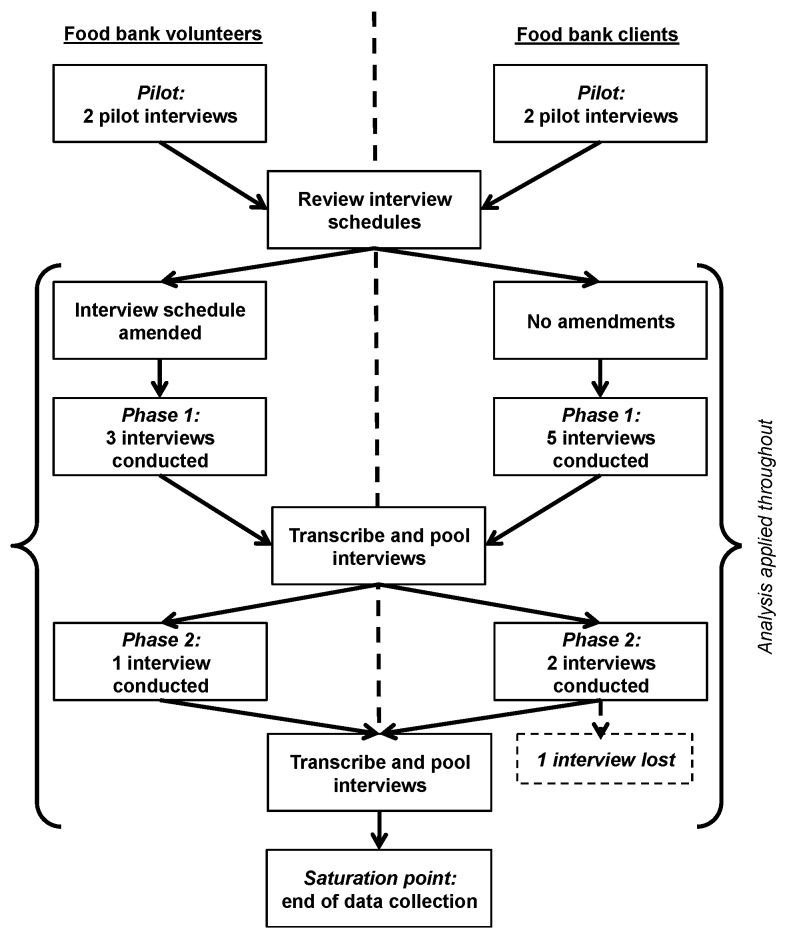
Flowchart of data collection and analysis.

To meet the study aims, triangulated interviews with food bank clients and volunteers were conducted to characterise sugar intake behaviours, unearth understanding and attitudes towards the health effects of sugar consumption, and gauge reaction to material from an information-based sugar reduction intervention called “Sugar Smart”. Qualitative methods were used because they enable a sensitive exploration of experiences, contexts and behaviours [40], and are valuable for intervention development [41]. Semi-structured, face-to-face interviews were conducted with eight food bank clients (C1–C8) and six volunteers (V1–V6, see Figure 1). Interviewees were purposively sampled from four Trussell Trust food bank outlets in Bristol, southwest England. In the years preceding this study, Bristol experienced rapid increases in relative deprivation, culminating in 16% (69,000) of residents living in the most deprived areas of England in 2015 [42]. The Trussell Trust is one of the main food bank organisations in Bristol, and they tripled their support for individuals experiencing food poverty between 2011/12 and 2013/14 [43].

After the food bank manager provided consent for data collection on these premises, the lead researcher (HF) made preparatory visits to each outlet where she displayed posters advertising the research. Recruitment began two weeks later, on a voluntary, first-come, first-serve basis, and entailed the researcher approaching clients and volunteers about partaking in the research. Inclusion criteria included being able to read English and, for clients, not using the food bank as their sole source of food supply to ensure some autonomy to food choices. The study was granted ethical approval by the Centre for Exercise, Nutrition and Health Sciences ethical committee at the University of Bristol (EAN 024-16), and all participants provided written consent.

### 2.2. Procedure

Interviews took place immediately after recruitment in quiet, public areas of each outlet to ensure interviewer and interviewee safety. Interviews were audio-recorded using an Olympus (DS-3500) encrypted digital recorder, and recruitment continued until theoretical saturation was reached for the entire sample through the repetition of responses [44] (see Figure 1). All interviews were transcribed verbatim, and an interview diary was used to collate observations about outlets and individual participants to support data interpretation [45]. Food bank clients received a £10 shopping voucher in gratitude for their participation, despite the risk that this might bias recruitment [46].

The interview guides were piloted with the first two clients (C1, C2) and volunteers (V1, V2), to ensure they would capture necessary information [47]. These interviews were incorporated into the main dataset for analysis. The interviews began by collecting some basic demographic information and then ice-breaker questions regarding food bank use, to develop rapport and encourage information sharing [48]. Subsequent questions addressed the distinct research enquiries by asking about factors influencing—and the perceived health effects of—food bank clients’ sugar consumption. To ensure common understanding across the sample, the researcher defined “added sugar” to interviewees as the sugar often added to products, rather than occurring naturally in foods, and commonly consumed in fizzy drinks, baked goods, or added to tea and coffee. 

The last section of the interview assessed the reception of an information-based sugar reduction intervention called Sugar Smart. Sugar Smart was an eminent information-based sugar reduction campaign being delivered throughout Bristol in 2017. Run by the chef and TV personality, Jamie Oliver, and Sustain, a food and farming advocacy organisation, the campaign was locally-delivered through partnerships with UK cities in which local authorities, organisations, workplaces and individuals pledged to become “sugar smart” [49]. Partway through the interview, participants were given a 7-page campaign leaflet, which contained information relating to the benefits of sugar reduction, the main sources of sugar, and advice on how to reduce sugar intake and read food labels. They were then asked about their understanding of the campaign and it’s appeal. To understand the extent that participants were basing their opinions of Sugar Smart on the leaflet presented in the interview, interviewees were asked what public health campaigns they were aware of, and whether they were aware of Sugar Smart prior to being given the leaflet to read. Throughout interviews, pre-planned and conversation-led probes were employed to delve deeply into any potentially relevant issues raised by participants [50].

### 2.3. Analysis

Data were thematically analysed, which entails examining meaning across the entire dataset while maintaining an interest in individual experiences [51]. This was achieved with a manual qualitative method: immersion in the data, coding, creating categories, and identification of themes [52]. The analysis process was repeatedly applied throughout data collection to systematically make sense of the whole dataset [53], ensure new data was effectively integrated [52], and identify the saturation point at which no new insights arose [54]. Analysis blended inductive and deductive reasoning, which is a well-accepted qualitative practice [55], by inductively identifying and defining each theme using the transcripts [51], and using deductive higher-order themes determined by the research aims and existing literature. The themes were organised into hierarchical frameworks, which help make social phenomena observed in the study generalisable to other settings [52]. Analysis was facilitated by NVivo computer software version 10.0 (QSR, Southport, UK).

The study was designed to adhere to qualitative research quality criteria and review guidelines [56,57]. This included triangulating interviews across the two interviewee groups, to provide a “stable view of ‘reality’” [58] (p7). Literature suggests that people tend to misreport their own dietary behaviours, particularly consumption of less healthy foods, like sugar [7]. Therefore, food bank volunteers were interviewed to triangulate with the views of food bank clients, because they have some interaction with the dietary choice of food bank clients, and thus may be able to provide valuable insights. Coding was also triangulated to minimise the influence of the researcher’s perspectives on resultant findings [59]. Referential adequacy was tested by initially archiving one transcript and then reintegrating it into the data set to test the validity of themes that had emerged [53]. A convergence coding matrix further improved credibility of the study findings, by facilitating comparison of evidence supporting higher order codes between interviewee groups [60].

## 3. Results

Characteristics of the interview participants are presented in Table 1. On average, interviews lasted 14 minutes, which includes the time spent reading the intervention leaflet. Household size ranged from lone living (C1, C6) to living with three others (C4, C7-8); with one interviewee disclosing that they did not have a permanent household (C2), and another that they lived in supported housing (C3). Generally, clients said their food bank use was infrequent: once every three-four weeks (C1, C2, C3, C6), or less frequently (C5: 3rd time in last seven months; C7: once every three-four months). For half of the clients interviewed, it was their second visit to the food bank (C1, C4, C6, C8).

### 3.1. Sugar Consumption Behaviours

**Figure 2:**
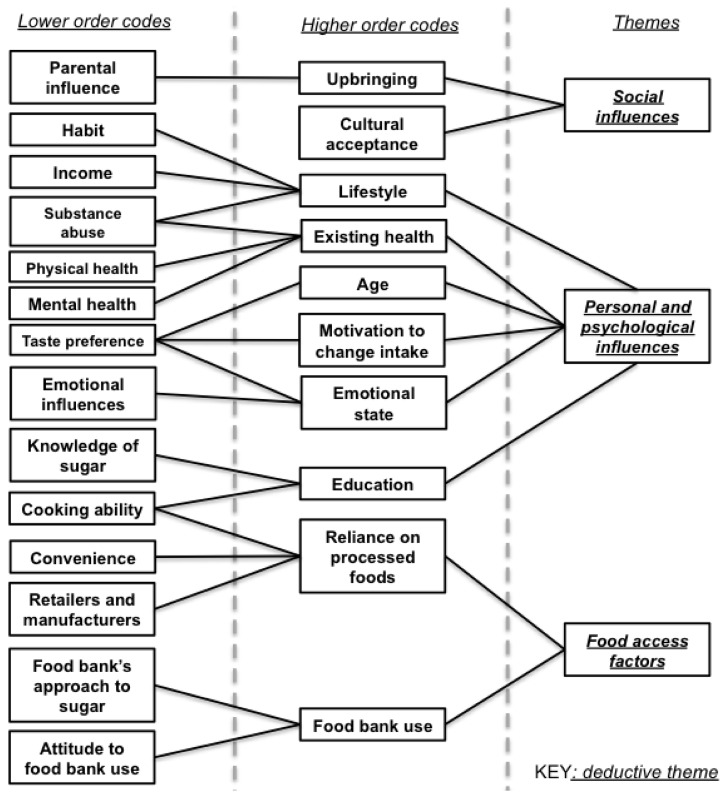
Hierarchical model of themes affecting sugar consumption behaviours.

Exploring the sugar intake behaviours of food bank clients began by characterising their consumption. Most clients felt their intake was high, with a couple saying they were *“terrible for sugar”* (C5, C8). While some volunteers were unsure of clients’ consumption behaviours, others referred to hot drink consumption habits in the food bank, and the behaviour of their own friends and family, to deduce that clients’ had heavy sugar consumption. Factors emerging to influence sugar consumption fell into three themes: personal and psychological influences, social influences and food access factors (Figure 2). 

#### 3.1.1. Personal and Psychological Influences

Personal and psychological factors appeared to influence food bank clients’ sugar consumption. Clients often referred to existing physical and mental health concerns in relation to their sugar consumption; with those reporting health concerns also tending to describe higher sugar consumption. Physical health concerns mentioned included alcoholism (*“my alcohol every day, that’s got a lot of sugar in it”;* C1), [suspected] diabetes, and obesity, while mental health concerns included attention deficit hyperactivity disorder, [symptoms of] depression, anxiety, addiction and other undisclosed concerns. A few clients associated addiction problems with their sugar consumption, as described by a client who was a former drug user: *“sweet stuff gives you a dopamine rush…like…taking drugs would…that’s why I think I turn to sweet stuff a lot”* (C3). Interestingly, sugar was also likened to drugs on several occasions (*“I just abuse sugar…my rush or something”,* C1; *“it’s like a bloody drug, innit’”,* C2; *it’s just like taking drugs”,* C3). 

In parallel to mental health concerns, food bank clients’ also reported that their emotional state influenced the amount of sugar they consume. Some associated sugar consumption with happiness (C5, C8) or making them feel better, though clients acknowledged that this depended on taste preferences *(“[I’m] more of a sweet person”, C5).* Others made explicit the reciprocal association between sugar consumption and how they were feeling, by referring to “*comfort eating”* (C3, V4). For some, this appeared to manifest as binge-like, disordered behaviour (*“[I] don’t even stop to think”* (C8)), which sometimes led to regret: *“I may have a chocolate bar… [I] go overboard on it if I feel really bad”* (C3); [after consuming 12 chocolate éclairs] *“I done the lot, and I was thinking ‘wow, I shouldn’t have done that’”* (C1). One volunteer felt that clients would be more likely to have this emotional tie to sugar consumption because of challenging circumstances they may be experiencing: *“people who are having a bad time will tend to turn to something of the comfort food variety a lot more”* (V4). A client reinforced this: “*I do turn to sugar in times of need”* (C3).

Other personal factors influencing sugar consumption included motivation. While some food bank clients were enthusiastic about reducing their sugar intake, clients and volunteers recognised that motivation might be affected by personal circumstance, particularly in relation to financial security: *“people who are in difficult positions in their lives, financially, feel a lack of control about everything in their lives, so they almost feel they can’t do anything about anything”* (V4). Similarly, a few clients said their attempts to monitor sugar consumption have increased with age, in part due to changing taste preferences (*“it’s just too sweet for me nowadays”,* C2). Notably, clients’ knowledge of sugar was also limited, which is could further promote consumption (*“I probably have sugar without even knowing that I’m actually having sugar*”, C1). Clients were confused about different types of sugar (*“I have not got a clue”,* C1; *“brown sugar isn’t as bad”,* C3), which may be affected by their educational attainment and conflicting terminology used in pre-made foods (*“that’s good for you though isn’t it, glucose?”* C1; *“unnatural sugars…is pumped with lots of extra bits”* C8). 

#### 3.1.2. Social Influences

Social influences also explained the sugar consumption of food bank clients. Clients referred to being *“brought up”* on sugar, with one associating their mother’s baking with present taste preferences: *“because my mum used to bake as well, I’m very used to eating cake”* (C3). Another client felt their living in a children’s home contributed to their dependence on sugar, as this left them unable to cook and so reliant on often sugar-heavy, premade foods (C1). Others also heavily consumed premade foods, but mainly for convenience given their other personal circumstances: *“I suffer anxiety and depression, and I eat a lot of rubbish and it makes me feel better…but then, like, I can’t be bothered then so I do things to be convenient”* (C8). One volunteer suggested that cultural acceptance of sugar consumption might promote these behaviours: *“I still think there’s an acceptance that sugar is OK”* (V3); leading them to believe: *“It’s impossible socially, to try and give up sugar”* (V3). 

#### 3.1.3. Food Access Factors

Food access also affected sugar consumption, though volunteers identified this more often than clients. Multiple clients and a volunteer remarked on finding sugar consumption unavoidable when noting the difficulty in accessing sugar-free foods: *“Everywhere you know, you will have sugar, no matter what…you have to be really good at finding something that doesn’t have sugar…the thing is, sugar’s in like every food stuff”* (C3). Volunteers suggested clients were more likely to consume processed foods because they are cheaper. This related to comments by a client: “*I suppose the food we buy, we haven’t really got a choice”,* C2). Volunteers expressed frustration with the conflict between the need to reduce sugar intake, and manufacturers’ and retailers’ behaviour: “*they’ve got all the like, chocolate stuff on the counter, why don’t they put fruit?”* (V6),*“food manufacturers ought to be trying to cut down more on the sugar”* (V1). Volunteers also felt food bank use contributed to eating behaviours, albeit they supported the food bank’s move to no longer provide bags of sugar in standard parcels: *“I think that’s probably a jolly good step…to take it away as being a necessity…make people aware…that it’s not a necessity”* (V5). However, the inherent difficulty with promoting better diet among clients dependent on non-perishable, processed items was highlighted: *“I mean we can kind of say ‘well we won’t stock this and we won’t stock that’, but we would end up not having anything on our shelves”* (V1).

### 3.2. Sugar and Health Understanding

**Figure 3:**
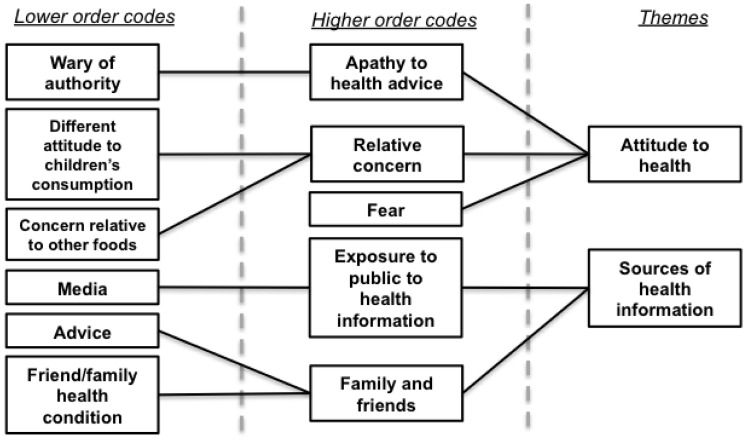
Hierarchical model of factors contributing to understanding the health effects of sugar consumption.

Participants demonstrated varied understanding of the health implications of over-consuming sugar. While some gave examples of diabetes, cancer, heart conditions, and dental problems, others revealed some confusion: *“It’s really bad for the blood, isn’t it? It can fur up your arteries”* (C3), and this appeared to manifest most in those reporting heavy sugar consumption. Factors affecting this understanding are shown in Figure 3.

#### 3.2.1. Sources of Health Information

Understanding the health effects of over-consuming sugar depended on the source of health information. Clients reported that family and friends provide them with health information, either through direct advice or because of their own health conditions that food bank clients’ felt were a direct consequence of heavy sugar consumption. Exposure to public health information was also important, particularly through their engagement with specific media sources. Clients mainly heard about the health effects of sugar and public health campaigns through television or *“the news”,* whereas volunteers referred to libraries, magazines, and newspapers. When asked to recall public health campaigns, some referred to smoking cessation campaigns or generic healthy eating or exercise promotion messages, but few could recall anything distinct. One volunteer correctly identified Change4Life. Excepting C6, all interviewees called for more education and awareness-promotion of the health effects of heavy sugar consumption. 

#### 3.2.2. Attitude to Health

Clients’ health attitudes also influenced how they perceived over-consuming sugar. For some, this was due to their concern for other foods, although they were similarly unclear of those health implications: “*I’m more cautious of salt…’cos I think salt ‘your heart’”* (C1). One client appeared to have greater concern about their children’s sugar consumption than their own *(“if it’s got…loads of sugar, I’m like you’re not having it”,* C7). Feeling apathetic towards their health also contributed towards food bank clients’ perception of the impact of sugar consumption, as expressed by three clients and two volunteers. This appeared somewhat attributable to wariness of authority, expressed by one client’s reluctance to speak with their doctor: *“Cos I’m one of them people that like, I put it aside. If I’m scared of something, I’ll be like ‘nah, it’s alright’”* (C1). Despite this, some clients voiced fear of the potential health implications of sugar consumption, promoted by an array of factors that ranged from knowledge gained in a previous job as a personal trainer (C3), to a recent experience of collapsing that they suspected was caused by diabetes: *“It did scare me when I collapsed…it made me like…a sinking feeling in your heart”* (C1). 

### 3.3. Intervention Reception

**Figure 4:**
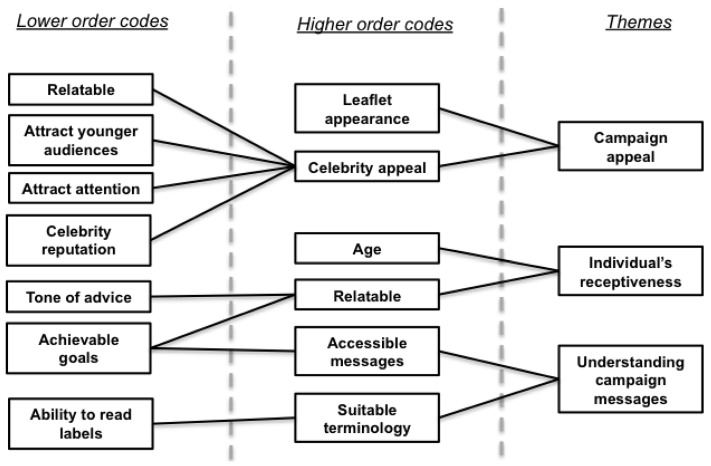
Hierarchical model of themes relating to Sugar Smart reception.

Only six interviewees said they had heard of Sugar Smart before the interview (43%); albeit the sources that interviews cited made it unclear whether the campaign had been correctly identified (smartphone apps, word-of-mouth, TV advertisements, and their doctor’s surgery). Figure 4 illustrates factors affecting their reception of the campaign based on the leaflet used in interviews.

#### 3.3.1. Campaign Appeal

Participants positively associated celebrity involvement with campaign appeal. They felt it was likely to make a *“massive, massive difference”* (C3), with Jamie Oliver preferable to other potential campaign figureheads because he was relatable: *“It would turn your head a bit more to listen or look”* (C8); *“getting to people who wouldn’t otherwise listen to an expert in a white coat”* (V5). One volunteer described specific characteristics contributing to this (*“[they don’t] have a really posh voice!”,* V4), and the fact they appeal to young people, emphasising the risks of selecting a celebrity too remote that might make clients think: *“oh well, it’s OK for you”* (V3-4). Participants described such “relatability” further: *“[People] look up to the celebrities like, ‘oh they’re involved, I can be too’”* (C7); *“It has to be the right sort of person…not patronising obviously, but relatively simple and straightforward”* (V4). Celebrity reputation was also considered important to the intervention, with one client describing the celebrity as an *“icon”* (C3), and others feeling they could attract younger audiences: *“[They’re] trying really hard to get…children and young people to know and understand”* (V2).

#### 3.3.2. Understanding Campaign Messages

All interviewees were positive about the leaflet appearance, remarking that they liked the colouring and branding, and that it is *“eye-catching”* (C5). However, one client stated: “*I think the messages is more important than the colouring”* (C3). While clients felt the leaflet presentation made the messages accessible (*‘it’s clear…basic, quick information that gives out a lot of information on the topic”,* C7), this was limited by their ability to comprehend some technical terms used for sugar (C1, V3). Similarly, clients’ ability to enact the campaign messages depended on being able to understand food labels: *“even how to read it on the box, I don’t even know how to do that”* (C5); *“maltrose, dextrose, they put all this in food don’t they, and it’s on the label but it doesn’t say sugar”* (V1). This made a volunteer wary of advice overly reliant on food labels (V3), and led another to conclude that advice in teaspoon units (as in the leaflet), would be difficult to correlate with sugar content measurements used on food labels that use conventional units (V4).

#### 3.3.3. Individual’s Receptiveness

The campaign also had to have meaning on an individual level. This included setting achievable goals that would be easier to implement: *“it did make me think well, I could actually do that…even as I was still reading, “oh when you have a cup of coffee you could have one less [spoon of sugar]”, because that’s achievable”* (C8). Similarly, the tone of advice influenced an individual’s receptiveness to the campaign (*“you don’t spout off too much…then you get bored of it”,* C5; *“it’s not even that it’s in your face…angry”,* C8). Finally, increasing age positively affected campaign reception (*“if I was younger, I would have been like ‘no’”,* C1).

## 4. Discussion 

This research qualitatively explored sugar consumption among LSES individuals from the UK, to understand the factors that influence their sugar consumption behaviour, their knowledge of the associated health effects of sugar consumption, and reception of a local information-based intervention. In doing so, the research has provided understanding of personal, psychological, social, and food access influences that are unique to—or exacerbated among—this population. Dependency on unreliable health information sources or having apathetic attitudes toward their health, may have diminished concern for sugar consumption among some food bank clients. This is particularly concerning given the number that disclosed existing health conditions. The extent to which these factors may manifest in food bank clients is illustrated in Figure S1. Perhaps these unique considerations made it unsurprising that an information-based intervention needed to be relatable to this population, in order to be well received. Things that contributed to intervention reception included the choice of celebrity, appearance of intervention material, and clarity of messages.

This research clarified the extent and nature of personal and psychological influences on sugar consumption for LSES individuals. The findings suggested that existing physical health conditions like alcoholism and drug addiction may be related to sugar consumption. This is explained in wider literature, which has found alcoholics and abstinent opiate and alcohol addicts to have a higher preference for sweetness [61,62]. Evidence that food cravings are reinforced by endogenous opiate release reinforces the parallels between sugar and drug cravings [63,64], which were drawn by participants. While the reciprocal relationship between sugar consumption and mental health emerging in this study has been reported elsewhere [65], the study was able to extend this knowledge by identifying how such health issues manifest in sugar consumption behaviours; namely, emotional or disordered binge-eating. Given there are separate health-related quality of life implications attached to disordered eating [66], this reciprocal relationship with sugar consumption is particularly damaging. It also emphasises the importance of intervening in the “reinforcing” nature of poor health among LSES populations. Physical and mental health issues may be over-represented in food bank populations than in general LSES groups [36], and some participants disclosed distrust or fear of medical professionals. Nonetheless, many disclosed existing physical and mental health concerns, therefore, it is important that easily accessible, relatable and attractively presented information is readily available in a range of healthcare settings, such as pharmacies and doctors surgeries. Similarly, one of the clients expressed greater concern for their children’s health, more so than their own, therefore, providing information in childcare settings might encourage clients to engage with material, to the benefit of both themselves and their children.

Food access factors also affected sugar consumption, though sampling in a food bank may over-emphasise this factor. Nonetheless, retailers, manufacturers, financial circumstance, and a lack of cooking ability all promoted food bank clients’ perception that consuming sugar is unavoidable. Food bank clients seemed somewhat dependent on processed foods, that are often heavy in sugar, because they were deemed most affordable. Reportedly poor cooking skills may compound this dependency on processed foods, corresponding with other research that has found cooking skills, assessed by self-reported confidence, to be least prevalent among lower-SES groups [67]. However, this subjective measure of cooking ability should be treated with caution, given that previous research suggests LSES individuals suffer from low self-efficacy [68]. Furthermore, despite the food banks’ efforts to promote better dietary quality, their inherent reliance on processed foods may also serve to reinforce heavy sugar consumption. There are limits to the extent that information-based interventions can address these complex issues affecting food supply; nonetheless, they might help LSES individuals “access” lower sugar foods by sharing and emphasising achievable ways of low-sugar cooking.

Understanding of the health effects of sugar consumption was mixed and unclear which, in the context of Health Belief Model [28], might suggest participants’ health is adversely affected by failing to fully perceive the risks of sugar intake [28]. Food bank clients are known to have received less education and have minimal financial resource [36], compromising their health literacy [32], and potentially explaining why clients mainly relied on informal health resources. Similarly, previous evidence associating lower levels of trust in information sources with low-income groups was reiterated in the present study [69]. A lack of reliable information is compounded by health attitudes. For example, clients’ concern for sugar relative to other foods resonate with evidence of LSES populations’ fatalistic attitudes to health [31]. When interpreted in the context of the Health Belief Model, these findings suggest barriers posed by wariness to health authority, combined with low perceived severity of the health effects of over-consuming sugar, contribute to clients’ current behaviours. In turn, this emphasises that food bank clients may stand to benefit from ensuring that information-based interventions are easily understood. 

The aforementioned findings developed understanding of the context of Sugar Smart, while a leaflet was used to gauge intervention reception. Clients liked the tone of messages, which relates to existing understanding that the least intrusive public health interventions are usually the most acceptable [70]. However, given that agency suggests that more intrusive approaches can be more effective [25], further research is required to establish whether receptiveness translates into behavioural change. Being able to understand the campaign messages is integral to their potential efficacy, but so is their potential for application, which one volunteer felt was limited by using spoon measures in the recommendations. While spoon measures may be more easily understood, they are only likely to be directly relevant to hot drink consumption. Conversely, recommendations in grams might be a harder to concept to convey but would help individuals understand food labels. For these reasons, future interventions may wish to reflect carefully on their choice of units used in advice and recommendations. 

The campaign appeal also benefitted from the involvement of Jamie Oliver, who was admired for his reputation and “relatability”. While several mechanisms explain the relationship between celebrity and health behaviours [71], for this study Jamie Oliver appeared to promote self-conception, in which people follow advice from celebrities that are similar to how they perceive themselves; and attachment, whereby individuals form connections with relatable celebrities [72]. Participants also noted how this celebrity was likely to appeal to younger audiences, thus appeasing their heightened concern for children’s health. While these findings are only based on the involvement of one celebrity, these mechanisms point more generally towards the importance of information-based campaigns involving celebrities acceptable to LSES populations. 

These insights have culminated in a number of recommendations for future, similar interventions, which are summarised in Table 2.

### Strengths and Limitations

To the authors’ knowledge, this is the first study to explore how an information-based sugar reduction intervention might influence the consumption behaviours of LSES individuals in this context. Importantly, this study also provided a voice to a population often under-represented in public health research [72]; and found that such “hard-to-reach” individuals usually participated enthusiastically. Given the on-going ambition to narrow health disparities, future research should make particular efforts to include such views. 

Nonetheless, the study encountered several limitations. Conducting interviews in public areas of food bank outlets may have accentuated social desirability bias [73], which is particularly relevant to sugar consumption research [74]. Food bank clients’ experiences of multiple deprivation and vulnerability may have increased risk of acquiescence bias [75]; and use of vouchers may have biased recruitment towards those in most financial struggle. Analytically, the researcher’s known interest in food banks may have introduced researcher bias [76], by being overly sympathetic to clients’ experiences. The experiences of clients may not generalise to all LSES populations [77], nor are they a representative subsample of all those who are food insecure across the UK. Limitations of volunteer perspectives should also be acknowledged, since they were likely to be overly interested in the dietary choices and wellbeing of clients. Volunteers may also be making assumptions about food banks clients sugar consumption which, combined with their attitudes, could bias the results. Overall, this suggests that further research could engage a wider range of LSES perspectives to confirm findings emerging in the present study. While attempts have been made to produce generalisable recommendations for other sugar reduction interventions, it is worth remembering that findings from this single intervention involving one celebrity may not correspond to other campaigns to limit sugar consumption. Finally, since there are known limitations to printed, untailored materials for nutrition education akin to the campaign leaflet used in this study [78], these findings may not generalise to the other facets of the campaign, which could be explicitly explored in future research.

## 5. Conclusions

Upstream interventions may remain most likely to effectively and equitably reduce sugar consumption. Nonetheless, it is also likely that policymakers will continue to pursue information-based sugar reduction interventions. This research provides valuable recommendations on how to ensure these interventions are well received by LSES populations, who could benefit most from reduced sugar consumption. Further research could attempt to quantify the impact of information-based interventions on the behaviours of these individuals. Given that we know there is no “silver bullet” to sugar reduction [4], future research could also develop understanding of how interventions across the agency-spectrum should work in synergy [25]. No doubt sugar reduction remains challenging, but ultimately worthwhile: *“it’s gonna’ be a massive undertaking…‘cos it’s life-long habit - not everyone’s a smoker but everyone’s an eater, and a drinker as well!’”* (C3). 

## Figures and Tables

**Table 1:** Demographic characteristics of participants.

Participant Characteristic		Clients (*n* = 8)		Volunteers (*n* = 6)		TOTAL (*n* = 14)	
		*n*	%	*n*	%	*n*	%
**Gender**	Male	3	38%	1	17%	4	29%
	Female	5	63%	5	83%	10	71%
**Age**	18–24	1	13%	-	-	1	7%
	25–34	2	25%	-	-	2	14%
	35–44	2	25%	-	-	2	14%
	45–54	2	25%	1	17%	3	21%
	55–64	1	13%	2	33%	3	21%
	65+	-	-	3	50%	3	21%
**Ethnicity**	White British	6	75%	6	100%	12	86%
	Black British	1	13%	-	-	1	7%
	Black Caribbean	1	13%	-	-	1	7%
**Household size**	Lone living	2	25%	-	-	-	-
	3 persons	1	13%	-	-	-	-
	4 persons	3	38%	-	-	-	-
	Other	2	25%	-	-	-	-

**Table 2:** Recommendations for information-based sugar reduction interventions.

Intervention characteristic	Recommendation
Partnerships	1. Engage more celebrities relatable to LSES populations
	2. Work with food providers in deprived areas
Distribution	3. Intervene in a range of healthcare settings
	4. Intervene in childcare settings
	5. Conduct more campaign activity in deprived areas
Material	6. Emphasise achievable methods of low-sugar cooking
	7. Consider using gram measurements in published material
	8. Consider adapting material to younger audiences

## Supplementary Materials

The following are available online at http://www.mdpi.com/1660-4601/16/3/413/s1, Figure S1: Themes manifesting in one participant.
