# Supplementary figures and images for: A Qualitative Study to Understand the Potential Efficacy of an Information-Based Sugar Reduction Intervention among Low Socioeconomic Individuals in the UK
Source: Int J Environ Res Public Health. 2019 Jan 31;16(3):413. doi: 10.3390/ijerph16030413 (PMC6388364; doi:10.3390/ijerph16030413)

Figure S1: Themes manifesting in one participant

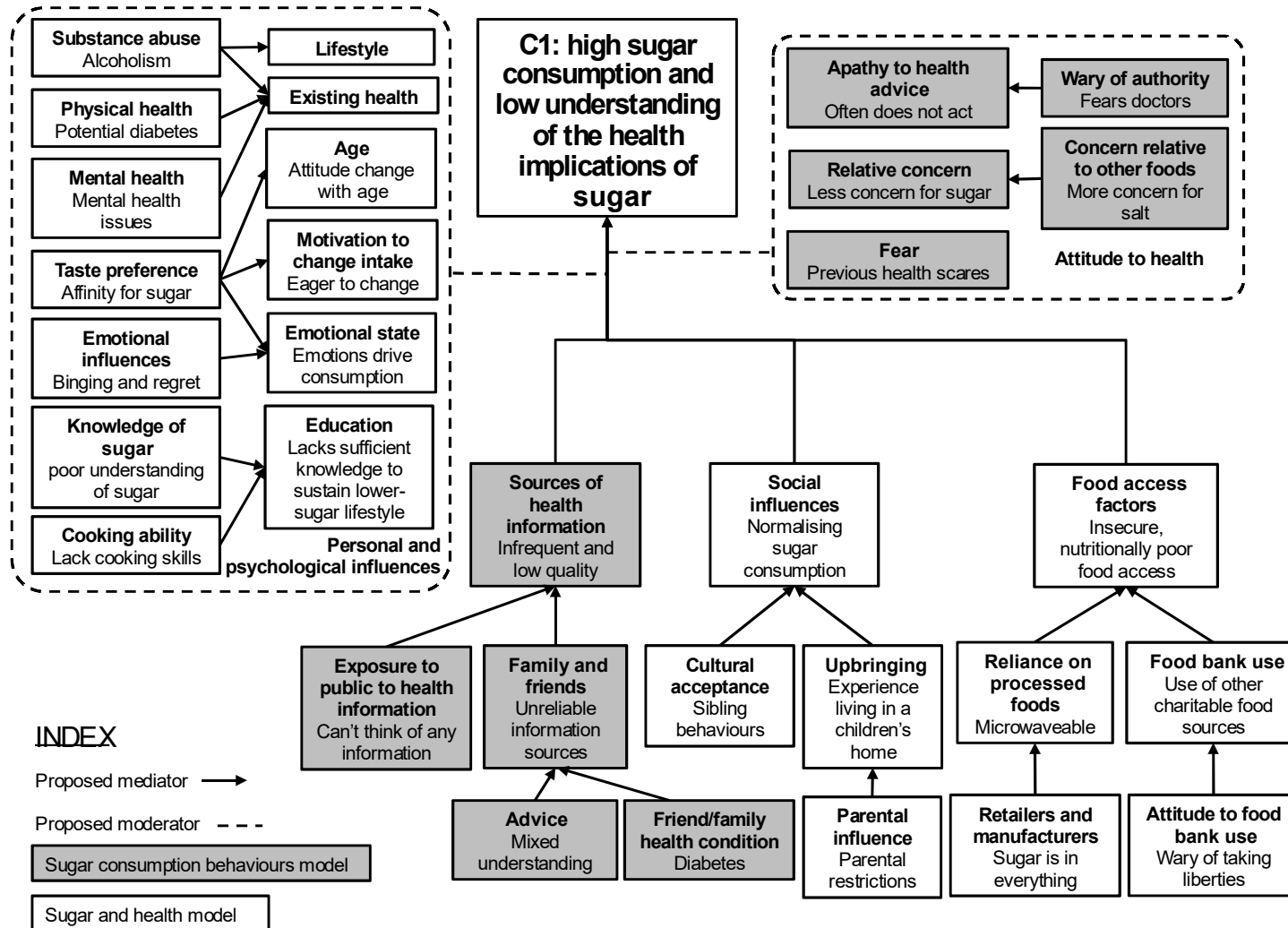

Supplement: Supplementary file 1 [file ijerph-16-00413-s001.pdf]
